# Comparative analysis of the *Dicer*-like gene family reveals loss of miR162 target site in *SmDCL1* from *Salvia miltiorrhiza*

**DOI:** 10.1038/srep09891

**Published:** 2015-05-13

**Authors:** Fenjuan Shao, Deyou Qiu, Shanfa Lu

**Affiliations:** 1 Institute of Medicinal Plant Development, Chinese Academy of Medical Sciences and Peking Union Medical College, Beijing, China; 2 State Key Laboratory of Tree Genetics and Breeding, The Research Institute of Forestry, Chinese Academy of Forestry, Beijing, China

## Abstract

*DCL1*, the core component for miRNA biogenesis, is itself regulated by miR162 in *Arabidopsis*. MiRNA-mediated feedback regulation of *AtDCL1* is important to maintain the proper level of *DCL1* transcripts. However, it is unknown whether the miRNA-mediated regulation of *DCL1* is conserved among plants. We analyzed the *SmDCL* gene family in *Salvia miltiorrhiza*, an emerging model plant for Traditional Chinese Medicine (TCM) studies, using a comprehensive approach integrating genome-wide prediction, molecular cloning, gene expression profiling, and posttranscriptional regulation analysis. A total of five *SmDCLs* were identified. Comparative analysis of *SmDCLs* and *AtDCLs* showed an apparent enlargement of *SmDCL* introns in *S. miltiorrhiza*. The absence of miR162 in *S. miltiorrhiza* and the loss of miR162 target site in *SmDCL1* were unexpectedly found. Further analysis showed that the miR162 target site was not present in *DCL1* from ancient plants and was gained during plant evolution. The gained miR162 target site might be lost in a few modern plants through nucleotide mutations. Our results provide evidence for the gain and loss of miR162 and its target sites in *Dicer*-like genes during evolution. The data is useful for understanding the evolution of miRNA-mediated feedback regulation of *DCLs* in plants.

Small RNAs are noncoding RNAs of about 20−24 nucleotides in length. They play vital roles in multiple developmental and physiological processes in various organisms through sequence-specific regulation of target genes at the transcriptional or post-transcriptional level^1^. Based on the biogenesis pathways, plant small RNAs can be classified into two major classes, microRNAs (miRNAs) and small interfering RNAs (siRNAs). SiRNAs are a large small RNA class with four subclasses, including heterochromatic siRNAs (hc-siRNAs), *trans*-acting siRNAs (ta-siRNAs), natural antisense transcript-derived siRNAs (nat-siRNAs), and long siRNAs (lsiRNAs)^2^. MiRNAs are produced from transcripts with internal stem-loop structures^3^, whereas plant siRNAs are derived from inverted repeat sequences, dsRNAs copied from single-stranded RNAs (ssRNA), over-lapping regions of bidirectional transcripts, or dsRNAs formed by virus replication^4^. Plant small RNAs regulate gene expression by loading into RNA-induced silencing complexes (RISCs) and then interacting with homologous RNA or DNA molecules for direct RNA cleavage, translational repression, or DNA methylation. The biogenesis and function of plant small RNAs involves various families of proteins, such as Dicer-likes (DCLs), HYPONASTIC LEAVES1 (HYL1), C2H2 Zn-finger protein SERRATE (SE), HEN1, HASTY, RNA dependent RNA polymerases (RDRs) and Argonautes (AGOs), of which DCLs are the core components for small RNA biogenesis^5^,^6^.

DCLs are multidomain ribonucleases characterized by six domains, including DExD-helicase (DExDc), helicase-C (HELICc), Duf283, PAZ, RIBOc and double stranded RNA-binding (dsRB) domain^7^. DExDc and HELICc existing in the N- and the C-terminals of the helicase region, respectively, are involved in ATP-dependent RNA or DNA unwinding. The ATP-binding site locates in DExDc domain. PAZ binds single-stranded RNAs with the two-base 3′-overhangs^8^. The RIBOc domain, known as ribonuclease III C terminal domain, is involved in dsRNA cleavage^9^, whereas dsRB mediates the discrimination of different RNA substrates and subsequent incorporation of effector complexes^7^. The function of DUF283 is currently unknown.

DCLs are usually encoded by a multiple gene family in plants. The number of *DCL* genes in each plant species may be varied. For instance, there are four in *Arabidopsis*^10^, five in poplar, maize and sorghum^11^, seven in tomato^12^, and eight in rice^13^. Among them, *Arabidopsis DCLs* (*AtDCLs*) are well-studied. Each of the four *AtDCLs* is primarily associated with the biogenesis of specific small RNA species, but they may play redundant and hierarchical roles in the production of various sRNAs^14^. *AtDCL1* is a core component for miRNA biogenesis, whereas *AtDCL2*, *AtDCL3* and *AtDCL4* are mainly involved in the derivation of siRNAs^15^. *AtDCL2* generates 22 nt siRNAs from endogenous inverted-repeats, integrated viruses and transgenes and plays significant roles in virus resistance and transitive silencing of transgenes^14^,^16^. *AtDCL3* is responsible for the derivation of heterochromatic siRNAs mostly from repetitive DNA loci. These siRNAs are about 24 nt in length and mediate the establishment and maintenance of heterochromatin states through RNA-dependent DNA methylation and histone modification^17^. AtDCL4 functions in the biogenesis of 21 nt phased siRNAs and ta-siRNAs^18^. It is also involved in dicing integrated viruses or transgenes into 21 nt siRNAs, which initiate transgene silencing and virus resistance^16^. These primary siRNAs may further initiate secondary siRNA production under the action of *AtDCL2*, *AtDCL4* and other genes^16^,^19^. In addition, the functions of various rice *OsDCL* genes have been analyzed. *OsDCL1* is involved in miRNA biogenesis as its *Arabidopsis* homolog, *AtDCL1*^20^. *OsDCL4*, the homolog of *AtDCL4*, is responsible for the biogenesis of 21 nt siRNAs associated with inverted repeat transgenes, ta-siRNAs and other 21 nt phased siRNAs, and has been found to play a broader role in rice development than *AtDCL4* in *Arabidopsis*^21^,^22^. *OsDCL3b*, rather than *OsDCL3a*, is involved in the processing of 24 nt phased siRNAs^22^. The function of *DCLs* in other plants is poorly understood.

It has been shown that *Arabidopsis AtDCL1* and *Physcomitrella patens PpDCL1* are negatively regulated by miRNAs. In *Arabidopsis*, miR162 target *AtDCL1* mRNA for direct cleavage at a complementary site formed by the splicing of exon 12 to exon 13^23^. Additionally, intron 14 of the *AtDCL1* primary transcript may form a hairpin structure generating *Arabidopsis* miR838^24^. Excision of *MIR838* precursor leads to the production of truncated, non-functional *AtDCL1* transcripts. It provides a regulatory feedback mechanism supplementing miR162-directed regulation to maintain the proper level of *AtDCL1* mRNA^24^. Similarly, intron 7 of the *PpDCL1* primary transcript forms a hairpin structure generating *P. patens* miR1047^25^. Although miR1047 and miR838 are different in sequence, generate from distinct intron number, and arise in an evolutionarily independent manner, miR1047 may play an analogous role of miR838 in the negative feedback regulation of *DCL1* in *P. patens*^25^. MiRNA-mediated negative feedback loops in other plant *DCLs* remain to be elucidated.

*Salvia miltiorrhiza*, which has been widely used for treating dysmenorrhoea, amenorrhoea and cardiovascular disease in China for thousands of years, is not only one of the best selling traditional Chinese medicine (TCM) but also an emerging model plant for TCM studies^26^,^27^,^28^,^29^,^30^,^31^,^32^,^33^ With the aim to elucidate the core components of gene silencing pathways in *S. miltiorrhiza*, we had previously identified the *SmAGO* and the *SmRDR* gene families^30^,^34^. Here we report the characterization of the *SmDCL* gene family using a comprehensive approach integrating genome-wide prediction, molecular cloning, gene expression profiling, and posttranscriptional regulation analysis. We showed the loss of miR162 target site in *SmDCL1* from *S. miltiorrhiza*. The results shed lights on the regulation and biological functions of *SmDCLs*.

## Results

### Identification and molecular cloning of five *DCL* genes in *S. miltiorrhiza*

**Figure 1:**
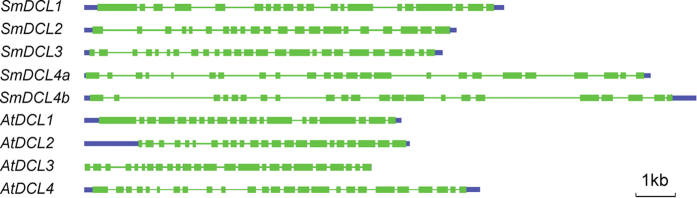
Gene structures of DCLs in S. miltiorrhiza and Arabidopsis. Exons are indicated in green boxes. UTRs are shown in blue boxes. Introns are indicated in lines.

Blast analysis of *Arabidopsis* and rice DCL amino acid sequences against the current assembly of the *S. miltiorrhiza* genome^31^ revealed five *SmDCL* loci (Fig. 1 and Table 1). Gene models were further predicted for 5 *SmDCLs* using Genscan (http://genes.mit.edu/GENSCAN.html)^35^ and corrected manually by comparison with *DCL* genes identified from other plant species using the BLASTx algorithm (http://www.ncbi.nlm.nih.gov/BLAST)^36^. The predicted *SmDCL* cDNAs encode proteins containing DExDc, HELICc, PAZ, dsRB and RNase III domains (Table 2), which are conserved in other plant DCLs, and show high sequence similarity with known plant *DCLs*, such as *AtDCLs* and *OsDCLs*, at both the nucleotide and amino acid levels. In order to validate the prediction of *SmDCL* genes, we cloned and sequenced the 5′ and 3′ ends of *SmDCL* cDNAs using RNA ligase-mediated rapid amplification of 5′ (5′ RACE) and 3′ (3′ RACE) cDNA ends, respectively. Based on sequence of the cloned 5′ and 3′ ends and the predicted *SmDCL* cDNAs, we designed primers and then PCR-amplified and sequenced full-length *SmDCL* cDNAs. Comparison of the cloned *SmDCLs* and the predicted ones showed that the gene models of *SmDCLs* were correctly predicted, although a few single nucleotide discrepancies most probably caused by polymorphisms and RT-PCR errors were found between the cloned and the predicted sequences. The results provide five experimentally validated full-length cDNAs of *SmDCLs* for further systematic characterization. Based on the similarities between *SmDCLs* and *AtDCLs*, the five *SmDCLs* were named *SmDCL1*, *SmDCL2*, *SmDCL3*, *SmDCL4a*, and *SmDCL4b*, respectively. The cloned *SmDCL* cDNAs have been deposited in GenBank under accession numbers shown in Table 1.

### Comparative analysis of *SmDCLs* and *AtDCLs* in sequence features, gene structures and conserved domains

Analysis of the cloned *SmDCL* cDNA showed that the length of open reading frames (ORFs) of *SmDCLs* varied between 4,158 (*SmDCL2*) and 5,772 bp (*SmDCL1*), 5′ untranslated regions (UTRs) varied from 45 (*SmDCL4a*) to 328 bp (*SmDCL1*), while 3′ UTRs varied between 166 (*SmDCL4a*) and 454 bp (*SmDCL4b*) (Table 1). The size of deduced SmDCL proteins varies between 1385 (*SmDCL2*) and 1927 (*SmDCL1*) amino acids, the molecular weight (Mw) varies from 156.3 (*SmDCL2*) to 216.4 kDa (*SmDCL1*), and the theoretical p*I* varies between 6.01 (*SmDCL1*) and 7.10 (*SmDCL2*) (Table 1). These sequence features of *SmDCLs* are quite similar to those of *AtDCLs* (Table 1). For instance, all SmDCLs and AtDCLs have the theoretical p*I* of about 6–7. SmDCL1 and AtDCL1 are the largest among DCL proteins in *S. miltiorrhiza* and *A. thaliana*, respectively. Additionally, the overall size of SmDCL proteins is comparable with the corresponding AtDCLs (Table 1).

Alignment of the cloned *SmDCL* cDNA with the corresponding genomic sequence showed that the intron number of *SmDCLs* varied from 19 (*SmDCL1*) to 24 (*SmDCL3*) (Table 1). *SmDCL1* and *AtDCL1* contain 19 introns and have very similar exon patterns (Fig. 1). The similarity of intron number and exon patterns was also found for other *DCL* gene pairs from *S. miltiorrhiza* and *A. thaliana* (Fig. 1). It suggests the conservation of *DCLs* in *S. miltiorrhiza* and *A. thaliana*. Interestingly, we observed an apparent enlargement of *DCL* introns in *S. miltiorrhiza* compared with *Arabidopsis* (Fig. 1). The expansion of intron size is probably due to the proliferation of transposable elements (TEs) during evolution or domestication of *S. miltiorrhiza*. However, it is necessary to further investigate the characteristics of introns in *SmDCLs* for elucidating the actual mechanism of intron size expansion.

Multiple sequence alignment of the deduced amino acid sequences using T-Coffee^37^ showed various conserved regions among *SmDCLs* (see Supplementary Fig. S1 online). Search of the deduced SmDCL proteins for conserved domains against the NCBI Conserved Domain Database (CCD) revealed that SmDCLs contained DExDc, HELICc, PAZ, dsRB and RIBOc domains (Table 2). These domains located in the conserved regions identified using T-Coffee^37^ and were also found in animal, fungal and other plant DCL proteins, suggesting the conservation of DCLs in organisms.

### Phylogenetic tree construction for DCL proteins in *S. miltiorrhiza*, *Arabidopsis* and rice

**Figure 2:**
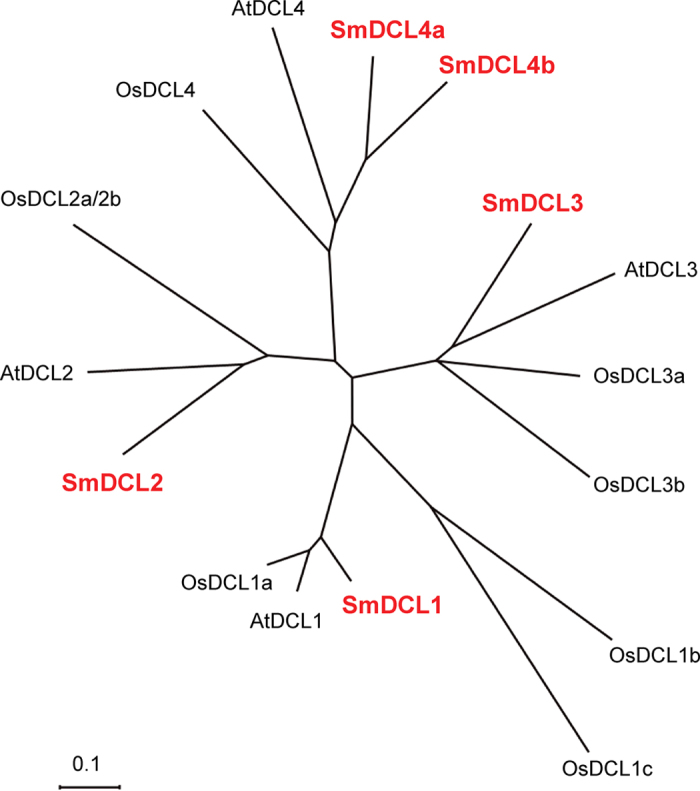
Phylogenetic tree of DCLs from S. miltiorrhiza, Arabidopsis and rice. The tree was constructed using MEGA 4.0 by the neighbor-joining (NJ) method with 1000 bootstrap replicates^38^,^59^.

An unrooted neighbor-joining (NJ) tree was constructed for determining the relationship of five SmDCLs, four AtDCLs and eight OsDCLs using MEGA4.0^38^ (Fig. 2). Based on the NJ tree, the referred DCL proteins fall into four clades. SmDCL1 clusters with AtDCL1, OsDCL1a, OsDCL1b and OsDCL1c in the DCL1 clade. SmDCL2 is closely related to AtDCL2, OsDCL2a and OsDCL2b in the DCL2 clade. SmDCL3, AtDCL3, OsDCL3a and OsDCL3b belong to the DCL3 clade. SmDCL4a and SmDCL4b cluster with AtDCL4 and OsDCL4 in the DCL4 clade. It suggests that each of four DCL clades include at least a SmDCL, implying the deeply conserved roles of SmDCLs with their counterparts in *Arabidopsis* and rice. Interestingly, two SmDCLs, including SmDCL4a and SmDCL4b, were found in the DCL4 clade. To our best knowledge, it is the first time to find two DCLs in a plant belonging to the DCL4 clade. *SmDCL4a* and *SmDCL4b* show similar exon patterns, whereas the size of various *SmDCL4a* and *SmDCL4b* intron is distinct (Fig. 1). For instance, introns 4, 5, 7, 15, 16 and 20 of *SmDCL4a* are longer than the corresponding introns in *SmDCL4b*, while introns 2, 8, 11 and 17 of *SmDCL4a* are apparently shorter (Fig. 1). It indicates that intron size expansion and condensation happened in *SmDCL4a* and *SmDCL4b*.

### Expression patterns of *SmDCL* genes in *S. miltiorrhiza*

**Figure 3:**
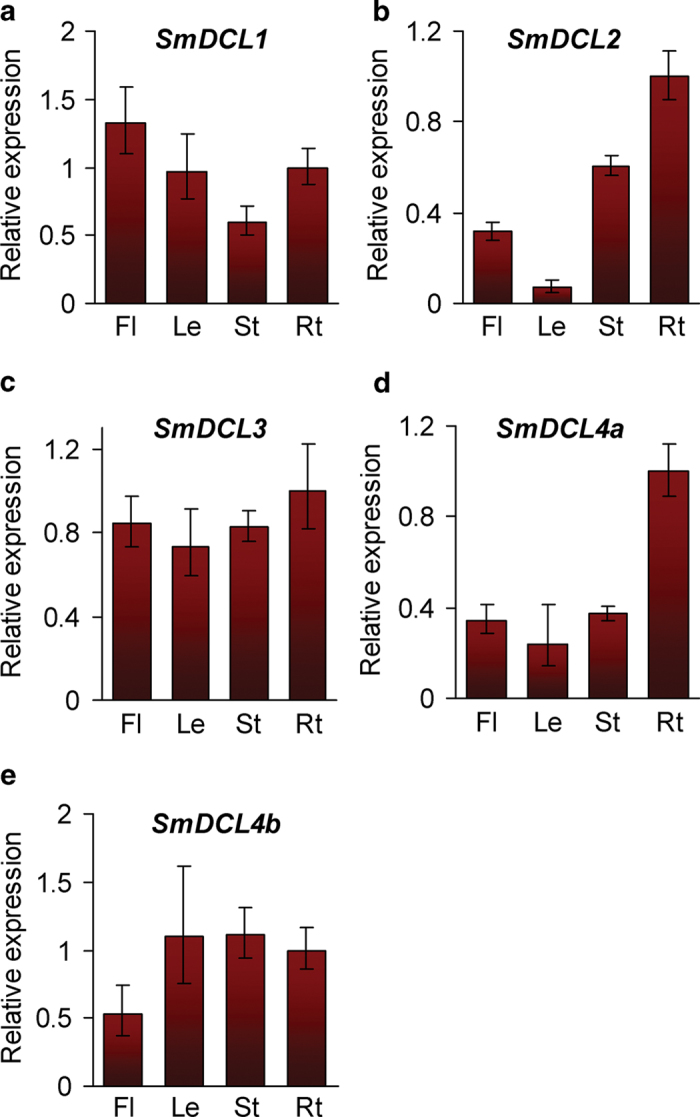
Expression of SmDCLs in flowers (Fl), leaves (Le), stems (St) and roots (Rt) of S. miltiorrhiza. Expression levels were quantified by qRT-PCR. The levels in roots were arbitrarily set to 1. Error bars represent the standard deviations of three technical PCR replicates.

As the core components for small RNA biogenesis, *DCLs* play vital roles in plant development^5^,^6^. The expression pattern of *DCLs* may be correlated with their physiological functions. With the aim to primarily elucidate the functions of *SmDCLs*, we analyzed the expression level of *SmDCL* genes in flowers, leaves, stems and roots of 2-year-old, field nursery-grown *S. miltiorrhiza* using quantitative RT-PCR technology. The results showed that all of five *SmDCLs* were expressed in *S. miltiorrhiza* tissues analyzed, although differential expression patterns were observed (Fig. 3). It is consistent with the significant role of *DCLs* in the biogenesis of miRNAs and siRNAs involving in plant development and stress responses. *SmDCL1* showed the highest expression in flowers, followed by roots and leaves, and less in stems (Fig. 3a). The pattern is very similar with that of its *Arabidopsis* counterpart, *AtDCL1*, which is consistent with the conserved roles of Sm*DCL1* and *AtDCL1* in miRNA biogenesis. The expression pattern of *SmDCL2* is similar with that of *SmDCL4a* showing more root-specific (Fig. 3b,d). Consistently, all of their *Arabidopsis* counterparts, *AtDCL2* and *AtDCL4*, are involved in transgene silencing and virus resistance, although *AtDCL2* generates 22 nt siRNAs, while *AtDCL4* functions in the biogenesis of 21 nt siRNAs^16^. It is noticed that *SmDCL4b* show a distinct expression pattern with *SmDCL2* and *SmDCL4a* (Fig. 3e), although *SmDCL4a* and *SmDCL4b* cluster in a clade (Fig. 2) and have similar exon patterns (Fig. 1). It indicates that *SmDCL4a* and *SmDCL4b* may play different roles in the production of siRNAs. Functional divergence of *DCLs* within a clade was previously found for rice *OsDCL3a* and *OsDCL3b*^22^. Both *OsDCL3a* and *OsDCL3b* belong to the DCL3 clade, whereas the processing of 24 nt phased small RNAs requires *OsDCL3b* rather than *OsDCL3a* in rice^22^. In *S. miltiorrhiza*, only one *SmDCL3* was identified. It expressed in flowers, leaves, stems and roots of *S. miltiorrhiza* at the similar levels (Fig. 3c), which seems to be consistent with the role of *DCLs* in the DCL3 clade, such as *AtDCL3* and *OsDCL3b*, in the derivation of heterochromatic siRNAs^17^.

### Analysis of miRNA-mediated regulation of *SmDCLs*

**Figure 4:**
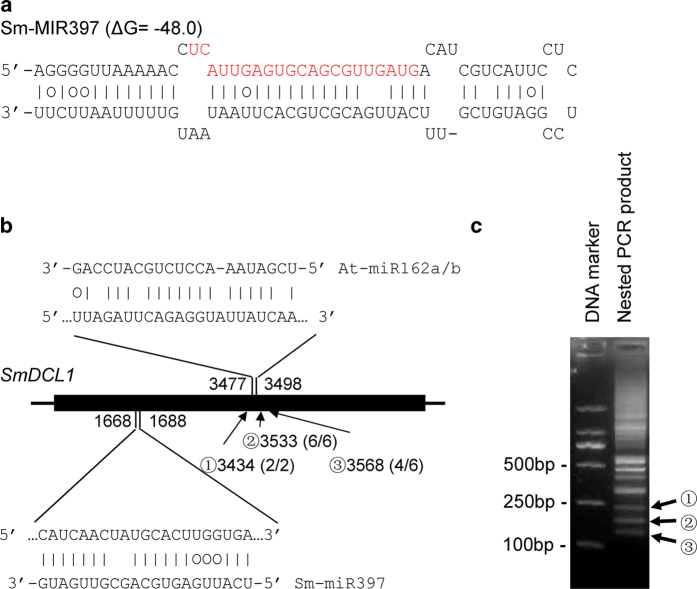
The Sm-MIR397 precursor and complementarities between miRNAs and SmDCL1. (**a**) Predicted hairpin structures of *Sm-MIR397*. Mature miRNA sequences are indicated in red. Vertical lines indicate G:C and A:U pairings. Circles indicate G:U pairings. (**b**) Complementarities between Sm-miR397, At-miR162a/b and SmDCL1. The heavy black line represents ORF. The lines flanking ORF represent nontranslated regions. MiRNA complementary sites with the nucleotide positions of *SmDCL1* cDNA are indicated. The RNA sequence of each complementary site from 5′ to 3′ and the predicted miRNA sequence from 3′ to 5′ are shown in the expanded regions. Arrows indicate the 5′ termini of three cDNA fragments (**c**) with the frequency of clones (in parentheses) and the nucleotide positions of SmDCL1 cDNA shown. (**c**) Determination of the 5′ termini of truncated SmDCL1 cDNA fragments using the 5′-RACE method. Nested PCR products were separated in a 2% agarose gel.

*Arabidopsis AtDCL1* and *P. patens PpDCL1* involved in miRNA biogenesis are themselves regulated by miRNAs^23^,^24^,^25^. *AtDCL1* is directly cleaved by miR162^23^. The level of *AtDCL1* mRNA is also affected by the excision of *MIR838* precursor from intron 14 of *AtDCL1* primary transcripts^24^. Similarly, the level of *PpDCL1* mRNA in *P. patens* cells is negatively regulated by the generation of miR1047 from intron 7 of *PpDCL1* primary transcripts^25^. In order to know whether there is a miRNA-mediated feedback regulation of *SmDCL1*, we first analyzed the secondary structure of all 19 introns in *SmDCL1*. No stem-loop structures meeting the widely used criteria for miRNA precursors were predicted^39^. We next performed a target search of plant miRNAs in miRBase against *SmDCL1* and the other four *SmDCLs* using psRNATarget^40^,^41^. With the maximum expectation of 3.0 applied in the target search, a total of 10 miRNA familes, including miR397, miR1035, miR1536, miR4395, miR4407, miR2873, miR5164, miR5247 and miR5303 were identified. Further alignment of these miRNA sequences with the current assembly of the *S. miltiorrhiza* genome using SOAP2 with two mismatches allowed^42^ and secondary structure prediction for genomic DNA fragments surrounding these miRNA sequences using the mfold program^43^ allowed us to identify a precursor for miR397 (Fig. 4a). No precursors were predicted for the other 9 miRNAs, indicating they could be not present in *S. miltiorrhiza*. The identified *S. miltiorrhiza* miR397 showed near-perfect complementarity to *SmDCL1* with a penalty score of 3.5^44^ (Fig. 4b). Using the modified 5′-rapid amplification of cDNA ends (RACE) method^44^, we tested whether *SmDCL1* were authentic targets of miR397. Unfortunately, no RACE products were obtained for the predicted cleavage after repeated experiments.

### Loss of miR162 target site in *SmDCL1* and lack of miR162 in *S. miltiorrhiza*

**Figure 5:**
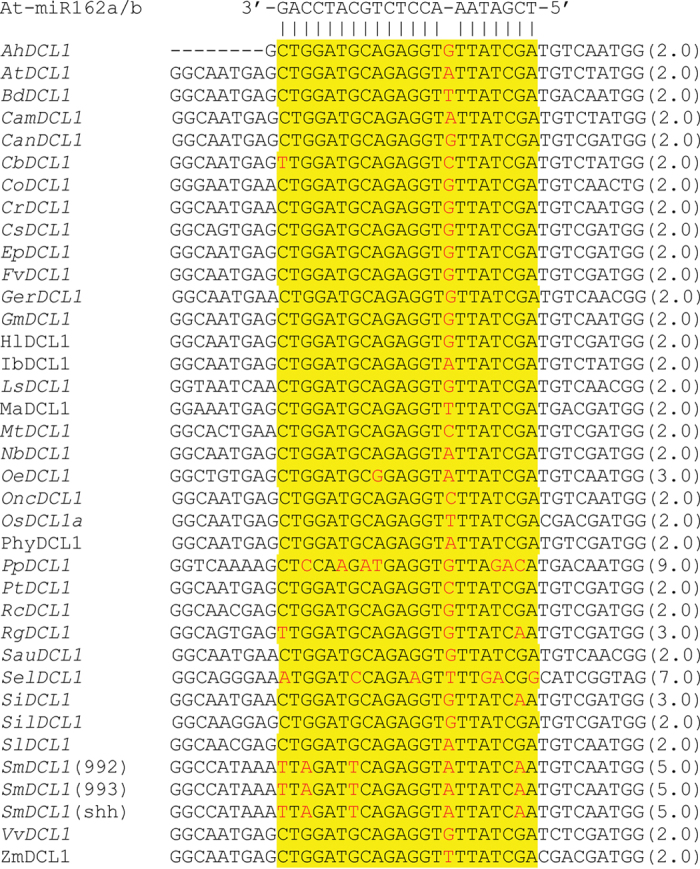
Alignment of the miR162 complementary site in DCL1s from various plant species. *A. thaliana* At-miR162a/b is also shown. Watson-Crick pairing is indicated by vertical dashes. Penalty scores for mismatched pattern in the miR162:*DCL1* duplex within a 20-base sequence window calculated as described previously are shown in parentheses (Lu *et al.* 2005). The sequences analyzed include *Arabidopsis* AtDCL1 (AT1G01040), *Arachis hypogaea* AhDCL1 (JR564267), *Brachypodium distachyon* BdDCL1 (XM_003558898), *Camelina sativa CamDCL1* (GABO01016802), *Cannabis sativa CanDCL1* (JP472773), *Catharanthus roseus CrDCL1* (GACD01069741), *Chorispora bungeana CbDCL1* (KA047874), *Chromolaena odorata* CoDCL1 (GACH01012147), *Cucumis sativus* CsDCL1 (XM_004155222), *Elaeocarpus photiniifolius* EpDCL1 (FX137492), *Fragaria vesca* FvDCL1 (XM_004308223), Gerbera hybrid cultivar GerDCL1 (GACN01020550), Glycine max GmDCL1 (XM_003553757), Humulus lupulus HlDCL1 (GAAW01068254), Ipomoea batatas IbDCL1 (JP112449), Lactuca serriola LsDCL1 (JO020520), Medicago truncatula MtDCL1 (XM_003558898), Musa acuminata MaDCL1 (JV351655), Nicotiana benthamiana NbDCL1 (KA746219), Olea europaea OeDCL1 (GABQ01046272), Oncidium ‘Gower Ramsey’ OncDCL1 (JL935168), Oryza sativa OsDCL1a (LOC_Os03g02970), Physalis peruviana PhyDCL1 (JO133983), Physcomitrella patens PpDCL1 (XM_001757896), Populus trichocarpa PtDCL1 (XM_002302643), Rehmannia glutinosa RgDCL1 (JG014336), Ricinus communis RcDCL1 (XM_002515051), Salvia miltiorrhiza line 993 SmDCL1(993) (KF366499), Salvia miltiorrhiza line 992 SmDCL1(992), Salvia miltiorrhiza line ssh SmDCL1(ssh), Saussurea involucrate SauDCL1 (JW888406), Selaginella moellendorffii SelDCL1 (XM_002965595), Sesanum indicum SiDCL1 (JP640291), Silene latifolia SilDCL1 (JO777655), Solanum lycopersicum SlDCL1 (10G005130), Vitis vinifera VvDCL1 (XM_002268333), Zea mays ZmDCL1 (DY397446).

In *Arabidopsis*, *AtDCL1* is an experimentally validated target of miR162^23^. However, it was not among the miRNAs predicted to target *DCLs* for cleavage in *S. miltiorrhiza*. Manual alignment of miR162 sequence from *Arabidopsis* with *SmDCL1* showed that the penalty scores for mismatched pattern in the miR162:*SmDCL1* duplex within a 20-base sequence window was 5.0 (Fig. 4b)^44^. Analysis of the target site variation between *A. thaliana* and *A. lyrata* for the highly conserved miRNA families showed that 10% of the *A. thaliana* miRNA-target pairs were lost^45^. In order to know whether the mismatched patterns of miR162:*SmDCL1* duplexes were conserved in different *S. miltiorrhiza* cultivation lines, we cloned *SmDCL1* cDNA fragments corresponding to the complementary sites of miR162 from the other two *S. miltiorrhiza* lines, namely 992 and shh. The result showed that the sequence of the complementary sites of miR162 in lines 992 and shh was identical to that in line 993 (Fig. 5), suggesting the conservation of the mismatched patterns of miR162:*SmDCL1* duplex in three lines of *S. miltiorrhiza* analyzed.

To test whether *SmDCL1* is regulated by miR162, the modified 5′-RACE analysis was carried out. After nested and nesting PCR amplification, at least ten cDNA bands were obtained (Fig. 4c). Sequence analysis of three cDNA bands with the approximately expected size showed that the 5′ end of PCR products located at upstream 52 bp, downstream 46 and 81 of the predicted cleavage site, respectively (Fig. 4b), suggesting they were not miR162-directed cleavage products.

Since the loss of miR162 target site in *SmDCL1*, we ask whether miR162 is present in *S. miltiorrhiza*. In order to address this question, we checked the published high-throughput sRNA sequencing data for mature miR162 sequence in *S. miltiorrhiza*^46^. No miR162 sequence was found in small RNA libraries for *S. miltiorrhiza* roots, stems and leaves. The read of miR162 sequence in flower small RNA library was only one. Extremely low small RNA reads could be a result from next-generation sequencing contamination^47^. To test this possibility, we first searched the current assembly of the *S. miltiorrhiza* genome for miR162 precursors. No positive results were obtained. Next, we searched our *S. miltiorrhiza* small RNA database for mature miR162 sequence. The database contains 114,426,648 clean reads obtained by high throughput Solexa sequencing of 18–30 nt small RNAs from flowers, leaves, stems and roots of *S. miltiorrhiza* plants. Consistently, no miR162 sequence was identified. Taken together, it is highly likely that miR162 is absent from *S. miltiorrhiza*.

### Mismatched patterns in the miR162:*DCL1* duplexes from 35 plant species

In order to know whether the absence of miR162-mediated feedback regulation of *DCL1* is widely present in plants or just limited to *S. miltiorrhiza* or a few plant species, an examination of the miR162 complementary site in *DCL1s* from 35 plant species was carried out. The cDNA regions complementary to miR162 are highly conserved among plant *DCLs*, except *SmDCL1*, *Physcomitrella patens PpDCL1*, *Selaginella moellendorffii SelDCL1, Rehmannia glutinosa RgDCL1*, *Sesamum indicum SiDCL1* and *Olea europaea OeDCL1* (Fig. 5). It suggests the conservation of miR162-mediated feedback regulation of *DCL1* in most plants. *PpDCL1* and *SelDCL1* with the penalty score for mismatched patterns in the miR162:*DCL1* duplexes to be 9.0 and 7.0, respectively (Fig. 5), have been confirmed to be not regulated by miR162^25^. The penalty score for miR162:*RgDCL1*, miR162:*SiDCL1* and miR162:*OeDCL1* duplexes is 3.0 (Fig. 5). No miR162 was found in more than 13 million unique sequences obtained by high throughput Solexa sequencing of 18–20 nt small RNAs from leaves, stems and roots of the first and second year cropping *R. glutinosa* plants^48^. Similarly, no miR162 was found in about 94 million sequence reads from juvenile and adult shoots, ripe and unripe fruits, and leaves of *O. europaea*^49^,^50^. It indicates that the miR162-mediated feedback regulation of *DCL1* seemed to be absent from *R. glutinosa* and *O. europaea*. The regulation of *SiDCL1* remains to be elucidated.

## Discussion

Although *DCLs* have been identified from various plant species, functional characterization of *DCLs* is limited to a few plants, such as *Arabidopsis* and rice^18^,^19^,^20^. The identification and molecular cloning of five *SmDCLs* provides a base for elucidating the function of *SmDCLs* and for understanding the biogenesis pathways and functions of small RNAs in *S. miltiorrhiza*, an emerging model plant with high medicinal value^26^. Five *SmDCLs* cluster into four clades with *Arabidopsis* and rice *DCLs* (see Supplementary Fig. S1 online), indicating the existence of four types of *DCLs* with distinct functions in *S. miltiorrhiza* as the cases in *Arabidopsis* and rice^7^,^13^,^51^. Conservation of sequence features, gene structures and functional domains implies that the function of each *SmDCL* could be similar to its *Arabidopsis* and rice counterparts in the same clade. However, it is interesting to show, for the first time, two *SmDCLs* in the DCL4 clade. *SmDCL4a* and *SmDCL4b* have similar exon patterns, but the intron size is distinct with some intron expanded while the others condensed (Fig. 1). Moreover, *SmDCL4a* and *SmDCL4b* showed distinct expression patterns (Fig. 3). These results indicate that *SmDCL4a* and *SmDCL4b* may play different roles in *S. miltiorrhiza* as the case of *OsDCL3a* and *OsDCL3b* in rice^22^. Further production and analysis of transgenic *S. miltiorrhiza* plants with *SmDCL4a* and/or *SmDCL4b* up- or down-regulated will definitely shed light on the biological function of *SmDCL4a* and *SmDCL4b*.

It has been shown the presence of miRNA-mediated feedback regulation of *Arabidopsis AtDCL1* and *P. patens PpDCL1*^23^,^24^,^25^. *AtDCL1* is regulated by miR162 and miR838^23^,^24^,^25^, while *PpDCL1* is regulated by miR1047^25^. Analysis of the regulation mechanism of *SmDCLs* unexpectedly revealed the loss of miR162 target site in *SmDCL1*. Close examination of the miR162 complementary regions showed the absence of miR162 target sites in *DCL1* from the non-vascular plant *P. patens* and the ancient vascular plant *S. moellendorffii*^25^,^52^, suggesting that the miR162 target site was not present in ancient plants and was gained during plant evolution. On the other hand, the gained miR162 target site might be lost in a few modern plants, such as *S. miltiorrhiza*. Since *S. miltiorrhiza* is evolutionarily far from *P. patens* and *S. moellendorffii* compared with many plants with the conserved miR162 target site (Fig. 5), gain and loss of miR162 target sites seems to be two independent events during plant evolution. Gain and loss of miRNA target sites has been previously investigated in *Arabidopsis* and rice^45^,^53^. The loss of miRNA target sites was proposed to be a consequence of gene ortholog loss, target site sequence disruption, or point substitutions/nucleotide mutations^45^,^53^. Analysis of the miR162 target sites (except the bulge nucleotide) showed single nucleotide mutation in *S. indicum SiDCL1* and *O. europaea OeDCL1*, two in *R. glutinosa RgDCL1*, while four in *S. miltiorrhiza SmDCL1* (Fig. 5). It suggests the loss of miR162 target sites was caused by nucleotide mutations rather than gene ortholog loss and target site sequence disruption.

It has been generally considered that miRNAs and their targets co-evolve in animals^54^. The absence of miR162 target site goes along with the lack of miR162 in *P. patens*^52^, *S. moellendorffii*^25^, *R. glutinosa*^46^, *O. europaea*^49^,^50^, and *S. miltiorrhiza*, suggesting that the miR162 gene, similar to the miR162 target site, might be lost in some modern plants during plant evolution, and indicating the possibility for co-evolution of miR162 and miR162 target sites in plants. However, since current information is preliminary, it is impossible to make a conclusion. Relatively frequent gain and loss of miRNA genes has been previously reported in *A. thaliana*^55^. Analysis of miRNA-target pair conservation between *A. thaliana* and *A. lyrata* showed that about 12.5% of non-conserved pairs were due to the loss of corresponding miRNAs in *A. lyrata*^45^. Of the 387 miRNAs from wild rice, 259 were not found in cultivated rice, suggesting a significant loss of miRNAs during rice domestication^56^. A possible mechanism for miRNA gene loss is nucleotide mutation. For instance, among 591 rice miRNAs, 364 have one or more SNPs in their precursor sequences^57^. SNPs in the stem regions may cause unstable of the miRNA hairpin structures, while SNPs in mature miRNAs have great potential to loss miRNA-target interaction^56^. Genome-wide duplication could be the other possible mechanism for the loss of miRNA genes. Comparative analysis of miRNA genes in maize and sorghum showed that duplicated miRNA genes underwent extensive gene-loss, with about 35% of ancestral sites were retained as duplicate homoeologous miRNA genes^58^. Since there is no information for miR162 gene variation among *S. miltiorrhiza* and its relative species and it is unknown for the genome-wide duplication events happened during *S. miltiorrhiza* evolution, the mechanism for loss of miR162 in *S. miltiorrhiza* is currently unknown and need to be further investigated.

It has been proposed that miR162-mediated feedback regulation of *DCL1* is important in maintaining *AtDCL1* at functionally sufficient, but not limiting or excessive, levels^23^, and the excision of *MIR838* precursor from *AtDCL1* primary transcript, which leads to the production of truncated and non-functional *AtDCL1* transcripts, provides a regulatory feedback mechanism supplementing miR162-directed regulation to maintain the proper level of *AtDCL1* mRNA^24^. Additionally, *P. patens* miR1047 seems to play a similar role in feedback regulation of *PpDCL1*^25^. However, data for the actual physiological functions of miR162, miR838 and miR1047 is lacking. Without direct physiological evidence, the significance of miRNA-mediated feedback regulation of *DCL1* is largely uncertained. The absence of miR162-mediated feedback regulation of *DCL1* in *S. miltiorrhiza* and probably in *R. glutinosa* and *O. europaea* implies that, at least in some plant species, miR162-mediated feedback mechanism could be not vital. It is possible that an alternative mechanism for maintaining *SmDCL1* at a proper level exists in *S. miltiorrhiza* and other plant species lacking the miR162-mediated feedback regulation of *DCL1*. Further investigating the regulatory mechanism of *SmDCLs* using transgenics may help to demonstrate the significance of miRNA-mediated feedback regulation of *DCL1* in plants and reveal the alternative of this feedback regulation in *S. miltiorrhiza*.

## Methods

### Plant materials

*S. miltiorrhiza* Bunge (line 993) was cultivated under natural growth conditions in a field nursery located at the Institute of Medicinal Plant Development, Beijing, China. Mature flower buds, mature and healthy leaves, young stems and roots in about 0.5 cm diameter were collected from two-year-old plants on August 15th, 2012. Tissues were collected from at least 3 plants and then pooled. The pooled tissues were stored in liquid nitrogen until use.

### Prediction and cloning of *SmDCL* genes

*SmDCL* genes were identified by tBLASTn analysis^36^ of *Arabidopsis* and rice DCL protein sequences (http://www.ncbi.nlm.nih.gov/protein) against the current assembly of the *S. miltiorrhiza* genome^31^. All retrieved DNA sequences were used for gene prediction on the Genscan web server (http://genes.mit.edu/GENSCAN.html)^35^. The predicted gene models were further examined and corrected manually by comparison with *DCL* genes identified from other plant species using the BLASTx algorithm (http://www.ncbi.nlm.nih.gov/BLAST)^36^.

To clone the full-length *SmDCL* cDNAs, RNA ligase-mediated rapid amplification of 5′ cDNA ends (5′-RACE) and 3′ cDNA ends (3′-RACE) was carried out using the GeneRacer kit (Invitrogen, Carlsbad, CA, USA). PCR amplification was performed using the following conditions: pre-denaturation at 94 °C for 2 min, 5 cycles of amplification at 94 °C for 30 s and 72 °C for 1 min, 5 cycles of amplification at 94 °C for 30 s and 70 °C for 1 min, 25 cycles of amplification at 94 °C for 30 s, 56 °C for 30 s and 72 °C for 2 min, followed by a final extension at 72 °C for 15 min. Nested PCR amplifications were carried out using the following conditions: pre-denaturation at 94 °C for 2 min, 30 cycles of amplification at 94 °C for 30 s, 58 °C for 30 s and 72 °C for 2 min, followed by a final extension at 72 °C for 15 min. PCR products were gel-purified, cloned and sequenced. The nesting and nested gene-specific primers used for 5′- and 3′-RACE are listed in Supplementary Table S1 and S2 online, respectively. Full-length *SmDCL* cDNAs were amplified using gene-specific forward and reverse primers (see Supplementary Table S3 online) under the following conditions: pre-denaturation at 94 °C for 2 min, 30 cycles of amplification at 94 °C for 30 s, 56 °C for 30 s and 72 °C for 3 min, followed by a final extension at 72 °C for 15 min. PCR products were gel-purified and cloned. For each transformation, three clones were sequenced at Beijing Sunbiotech Co., Ltd (Beijing, China). Sequences from three clones were aligned with the predicted *SmDCL* sequence using DNAMAN (Lynnon BioSoft, San Ramon, CA, USA). The cloned cDNAs showing the least nucleotide discrepancies with the predicted sequences were selected and deposited in GenBank (Table 1).

### Phylogenetic tree construction and bioinformatics analysis

Phylogenetic tree was constructed using MEGA version 4.0 by the neighbor-joining method with 1000 bootstrap replicates^38^,^59^. Intron/exon structures were analyzed manually based on genomic DNA sequences and the cloned cDNA sequences. Molecular weight (MW) and theoretical isoelectric point (p*I*) were predicted using DNAMAN. Conserved domains were analyzed by search the deduced amino acid sequence of *SmDCLs* against the NCBI conserved domain (http://www.ncbi.nlm.nih.gov/Structure/cdd/wrpsb.cgi). Multiple sequence alignment of the deduced SmDCL amino acid sequences was carried out using T-Coffee^37^.

### Quantitative real-time reverse transcription-PCR (qRT-PCR)

Total RNA was isolated from plant tissues using the plant total RNA extraction kit (BioTeke, Beijing, China) and genomic DNA was removed by treating with RNase-free DNase (Promega, Madison, WI, USA). One μg total RNA was converted into cDNA by 200 U Superscript III reverse transcriptase (Invitrogen, Carlsbad, CA, USA) in a 20 μl volume. cDNA was diluted into 200 μl and then used for qRT-PCR. Gene-specific primers were listed in Supplementary Table S4 online. *SmUBQ10* was used as a control as previously described^28^. PCR was carried out in a 20 μl volume containing 2 μl diluted cDNA, 250 nM forward primer, 250 nM reverse primer, and 1 × SYBR Premix Ex Taq II (TaKaRa Bio, Otsu, Japan) using the following conditions: pre-denaturation at 95 °C for 30 s, 40 cycles of amplification at 95 °C for 5 s, 60 °C for 18 s and 72 °C for 15 s. The results from gene-specific amplification were analyzed using the comparative Cq method, which uses an arithmetic formula, 2-ΔΔCq, to achieve results for relative quantification^60^. Cq represents the threshold cycle.

### Identification of *S. miltiorrhiza* miRNAs with perfect or near-perfect complementarity to *SmDCLs*

Plant miRNAs with the potential to target *SmDCLs* for cleavage were predicted using psRNATarget with the default parameters^40^. Known plant miRNAs were downloaded from miRBase (release 19, http://www.mirbase.org/)^41^. The identified miRNAs were then aligned with the current assembly of the *S. miltiorrhiza* genome^31^ using SOAP2 with no more than 2 mismatches allowed^42^. *S. miltiorrhiza* genomic DNA sequences with known plant miRNAs aligned were predicted for hairpin structures using mfold^43^. Criteria described by Meyers *et al*^39^ were applied to annotate *S. miltiorrhiza* miRNAs.

### 5′RLM-RACE for analysis of miRNA-directed cleavage of SmDCLs

The modified RNA ligase-mediated rapid amplification of 5′ cDNAs method (5′RLM-RACE) was performed using the GeneRacer kit (Invitrogen, Carlsbad, CA, USA) as described previously^44^. PCRs were carried out on mRNA isolated from pooled *S. miltiorrhiza* tissues containing flowers, leaves, stem and roots. Gene-specific primers used in this experiment are listed in Supplementary Table S5 online.

### PCR amplification of *SmDCL1* cDNA fragments in *S. miltiorrhiza* lines 992 and shh

*SmDCL1* cDNA fragments surrounding the predicted miR162 target site were PCR-amplified on cDNA from the leaves of *S. miltiorrhiza* lines 992 and shh using 5′-GTCAGGGAGGAGCTGTGACAATT-3′ as the forward primer and 5′-CGTACATGAAAGCTCTTGAGCGAT-3′ as the reverse primer.

## Additional Information

**How to cite this article**: Shao, F. *et al*. Comparative analysis of the *Dicer*-like gene family reveals loss of miR162 target site in *SmDCL1* from *Salvia miltiorrhiza.* *Sci. Rep.* **5**, 9891; doi: 10.1038/srep09891 (2015).

## Supplementary Material

Supplementary Information

## Figures and Tables

**Table 1:** Sequence features and intron numbers of SmDCLs and AtDCLs.

**Genename**	**Accession no.**	**No. of intron**	**ORF (bp)**	**5′UTR (bp)**	**3′UTR (bp)**	**Protein (aa)**	**MW (kDa)**	**PI**
SmDCL1	KF366499	19	5772	328	251	1927	216.4	6.01
SmDCL2	KF366500	20	4158	212	168	1385	156.3	7.10
SmDCL3	KF366501	24	4965	123	193	1654	184.3	6.76
SmDCL4a	KF366502	23	4887	45	166	1628	183.7	6.77
SmDCL4b	KF366503	22	4635	148	454	1544	175.0	6.17
AtDCL1	AT1G01040	19	5730	373	147	1909	213.6	6.16
AtDCL2	AT3G03300	23	4167	561	93	1388	156.9	6.77
AtDCL3	AT3G43920	21	4596	_	_	1531	172.0	6.23
AtDCL4	AT5G20320	24	5106	198	352	1702	191.3	6.74

**Table 2:** Location of conserved domains in SmDCL proteins.

**Protein name**	**DExDc**	**HELICc**	**dsRB1**	**PAZ**	**RIBOc1**	**RIBOc2**	**dsRB2/3**
SmDCL1	283–435	666–786	862–951	1218–1345	1384–1566	1603–1758	1854–1922
SmDCL2	33–178	366o–479	550–635	799–925	972–1116	1156–1309	1313–1377
SmDCL3	52–203	387–504	575–657	873–1005	1058–1218	1264–1413	-
SmDCL4a	44–241	377–508	567–646	827–956	1004–1175	1206–1351	1542–1616
SmDCL4b	58–209	371–508	564–646	841–954	1001–1158	1204–1350	1358–1425/1512–1544
