# Supplementary material for: Comparative analysis of the Dicer-like gene family reveals loss of miR162 target site in SmDCL1 from Salvia miltiorrhiza
Source: Sci Rep. 2015 May 13;5:9891. doi: 10.1038/srep09891 (PMC4429486; doi:10.1038/srep09891)
Supplement: Supplementary Information [file srep09891-s1.pdf]

Comparative analysis of the *Dicer*-like gene family reveals loss of miR162 target site in *SmDCL1* from *Salvia miltiorrhiza*

Fenjuan Shao[1,2], Deyou Qiu[2], Shanfa Lu[1,*]

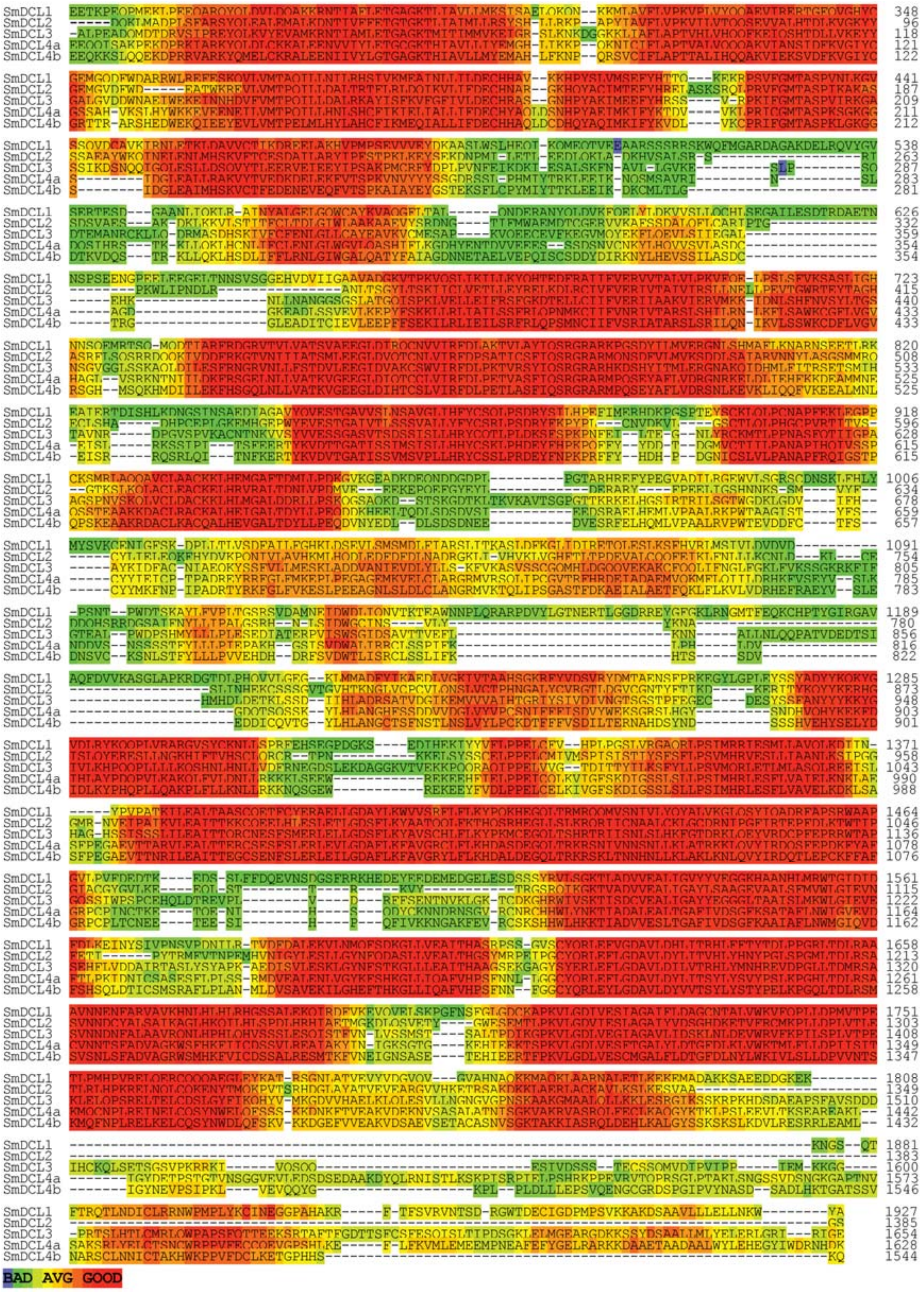

Supplementary Figure S2. Multiple sequence alignment of the deduced SmDCL amino acid sequences. The alignment was performed using T-Coffee. The best alignments are coded in red and the worst alignments are highlighted in blue.

Supplementary Table S1. Primers used for 5'-RACE of *SmDCLs*.

**Gene name**	**Primer sequence (5' to 3')**
SmDCL1	Nesting: GACACAGGCTTGCTGAGCAGAGGA
	Nested: CGAAGGTCTGCCATTAGCATCGA
SmDCL2	Nesting: GGTGAGAATAGCTGCGAAGAAGCA
	Nested: GAGAGCCATCTCTAGTGCTTCCAA
SmDCL3	Nesting: CCCTCCTCAAGCACATCAGTGGAA
	Nested: CGACACCAGAGTTACTTCCCGTCA
SmDCL4a	Nesting: CTATGACCTTGGCTTGCTGCTGAA
	Nested: CCAAAGCAACTGTAGGGGCAAGA
SmDCL4b	Nesting: CCTCTTCACCCACTTTGGTAGCAA
	Nested: GGAGAGGGATCTTGCAGTGGCAAT

Supplementary Table S2. Primers used for 3'-RACE of *SmDCLs*.

Gene name	Primer sequence (5' to 3')
SmDCL1	Nesting: GAGCGCAGAGGAGGATGATGGTA
	Nested: GGACTCTGCTGCAGTTCTTCTTCT
SmDCL2	Nesting: GGTGCATGAAGCCTCTGCTCGAT
	Nested: CAAGGAAAGCGTGGCTGCAGGTT
SmDCL3	Nesting: GCAATGGCGTTGGACCTAACTCGA
	Nested: GACCTCACTCCACACCCTATGCA
SmDCL4a	Nesting: CTGCCTCAGCTTTAGCTACCAACA
	Nested: GCTCCCACCAATGTATCGGCAAA
SmDCL4b	Nesting: GACCGGCATGAATTCAGAGCAGAA
	Nested: GCTTCCTGTTGTGGAGCACGACCAT

Supplementary Table S3. Primers used for amplification of full-length *SmDCL* cDNAs.

**Gene name**	**Primer sequence (5' to 3')**
SmDCL1	Forward1: CGATGCTAATGGCAGACCTTCGTA
	Reverse1: GATCAATGACTGCACCTTTGGAGT
	Forward2: CTCCAAAGGTGCAGTCATTGATCA
	Reverse2: GCATGTGCAGGTCCGCCCTCGTTT
SmDCL2	Forward1: GGAAGCACTAGAGATGGCTCTCAA
	Reverse1: CCAGTACAATGTTCTGAGGCTTCA
	Forward2: CAGAACATTGTACTGGCTGTCCAT
	Reverse2: CCTGCAGCCACGCTTTCCTTGAGA
SmDCL3	Forward1: GTTCGGGAAAGATACAGAGCTTCT
	Reverse1: GGAAGAGGTGGCAGCTAACGGCAT
	Forward2: GCCGTTAGCTGCCACCTCTTCCTT
	Reverse2: GTGCAGCAGAGTCGTACGAGCTCT
SmDCL4a	Forward1:CTTGCCCCTACAGTTGCTTTGGTT
	Reverse1: CAGCATCGGCAATCTCATCTCTAT
	Forward2: CATAGAGATGAGATTGCCGATGCT
	Reverse2:CTGCTGCTGTCTCGGCTGCATCCT
SmDCL4b	Forward1: GACACCTGAACTAATGTTGCACTA
	Reverse1: GCAGCCATTGGCAAGATGCAAGTA
	Forward2: CATCTTGCCAATGGCTGCACAAGT
	Reverse2: CGCGATTTGCATCTGGACCTTGTA

Supplementary Table S4. Primers used for qRT-PCR.

Gene name	Primer sequence (5' to 3')
SmDCL1	Forward: GCTCAGCAAGCCTGTGTCCAGGAT
	Reverse: CTCTCCTTTGGATCCCTTCCACGA
SmDCL2	Forward: GCACTAGAGATGGCTCTCAAAGAT
	Reverse: GAGGTCCGTATGTAGGGCCACAAC
SmDCL3	Forward: CAGTAATGCAGCGGCTGGAGACGC
	Reverse: GAAGAGGTGGCAGCTAACGGCATA
SmDCL4a	Forward: GGCCTTGGAGGAGAATGTCGTAA
	Reverse: CTATGACCTTGGCTTGCTGCTGAA
SmDCL4b	Forward: GTGGACAGGAGCAACCTCAAGGA
	Reverse: GTCACGTGGAAGTGATGAGCAATA
SmUBQ10	Forward: AGATGGGCGGACACTTGCTGATTA
	Reverse: ACTCTCCACCTCCAAAGTGATGGT

Supplementary Table S5. Primers used for analysis of miRNA-directed cleavage of *SmDCLs*.

**Gene name**	**miRNA name**	**Primer Sequence (5' to 3')**
SmDCL1	miR162	Nesting: GCATCTACAGATCTACTTCCAGT
		Nested: CCTTTGATGTGTCCCATGGAGTA
SmDCL1	miR397	Nesting: CTCCACCAGAGACAACTACCAT
		Nested: CCTCTAGCTCTTCAGGTCCAT
